# Effect of supplemental heat on mortality rate, growth performance, and blood biochemical profiles of Ghungroo piglets in Indian sub-tropical climate

**DOI:** 10.14202/vetworld.2016.396-402

**Published:** 2016-04-22

**Authors:** Hemanta Nath, Mousumi Hazorika, Dipjyoti Rajkhowa, Mrinmoy Datta, Avijit Haldar

**Affiliations:** 1 Animal Reproduction Division, ICAR Research Complex for North Eastern Hill Region, Tripura Centre, Agartala, Lembucherra, West Tripura, India; 2 ICAR Research Complex for NEH Region, Barapani, Umiam, Meghalaya, India

**Keywords:** biochemical profiles, Ghungroo piglets, growth, mortality rate, neonatal, supplemental heat

## Abstract

**Aim:**

The present study was conducted to explore the effect of supplemental heat on mortality rate, growth performance, and blood biochemical profiles of indigenous Ghungroo piglets in sub-tropical cold and humid climatic conditions of Tripura, a state of the north eastern hill (NEH) region of India.

**Materials and Methods:**

The experiment was conducted on 38 indigenous Ghungroo piglets from birth up to 60 days of age. Among the 38 piglets, 19 piglets were provided with supplemental heat ranging between 17.0°C and 21.1°C for the period of the first 30 days and thereafter between 24.1°C and 29.9°C for the next 30 days. The other 19 piglets were exposed to natural environmental minimum temperatures ranging between 7.2°C and 15.0°C during the first 30 days and then between 18.5°C and 25.5°C for the next 30 days.

**Results:**

The supplemental heat resulted in 10.6% reduction of piglet mortality from the 2^nd^ till the 7^th^ day of age. These beneficial effects could be related with the lower (p<0.05) plasma glutamate pyruvate transaminase (GPT) and cortisol levels and higher (p<0.05) plasma alkaline phosphatase (AP) concentrations in heat supplemented group compared to control group. Plasma AP, GPT, glucose, triiodothyronine, and luteinizing hormone concentrations decreased (p<0.05) gradually with the advancement of age in both control and supplemental heat treated piglets.

**Conclusion:**

Supplemental heat could be beneficial since it is related to a reduction of piglet mortality during the first week of life under farm management system in the sub-tropical climate of NEH region of India.

## Introduction

The survivability and growth of piglets are very important economic aspects for the success of pig farming. Piglet mortality during the perinatal and lactational period is one of the most crucial factors leading to reduced production efficiency in pig farming [1,2]. Besides, the economic losses, piglet mortality also represent a livestock welfare issue. The primary causes of live born piglet mortality are hypothermia, starvation, and crushing [3]. Newborn piglets are poorly insulated and lack of brown adipose tissue, and thus rely exclusively on shivering as the main mechanism for thermogenesis in the cold environment [4]. At birth, they usually experience a sudden drop of 2-4°C in the body temperature, and recovery of a normothermic temperature of 39°C is achieved after 24-48 h of life in adequate environmental condition [5]. However, excessive hypothermia due to severe environmental conditions, low body weight, or reduced vitality at birth could significantly reduce piglet vigor leading to the death of the animal [6]. Impairment of cellular immunity, another factor strongly related to piglet survival, is associated with overexpression of heat shock protein 70 in neonatal pigs [7].

Floor heating has favorable effects on the early recovery of piglet body temperature, latency to first suckle, and survival of piglets [8]. Straw can be used on the floor to provide warmth to the piglets during winter months. However, proximity to the straw is a concern with regard to increasing the risk of piglets crushing and enteritis [9]. Recent studies indicate that the maternal diet modulates the epigenetic regulation of hepatic gluconeogenic genes in neonatal piglets [10]. Limited studies concerning the provision of the warm environment to the newborn piglets and its beneficial effects on piglet’s survivability and performance have previously been performed with exotic pure or crossbred piglets in temperate climate [11]. However, there is no information on the effect of supplemental heat on the performance of indigenous piglets during winter in the Indian sub-tropical climate.

The aim of the present study was to explore the effect of supplemental heat on mortality rate, growth performance, and blood biochemical profiles of indigenous Ghungroo piglets during cold and humid weather in the north eastern sub-tropical region of India.

## Materials and Methods

### Ethical approval

The experimental protocol and animal care were in accordance with the National Guidelines for care and use of Agricultural Animals in Agricultural Research and Teaching.

### Study area

**Figure 1:**
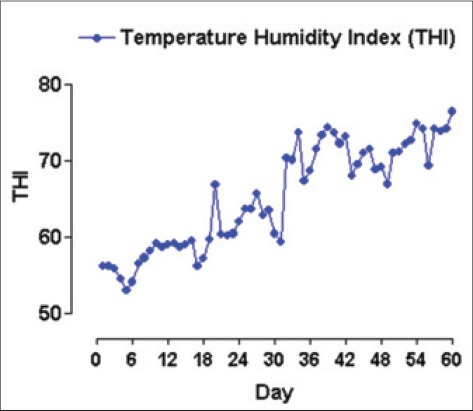
Daily temperature humidity index during the 60 days experimental periods (values ranged between 52.98 and 76.45).

The present study was conducted at pig farm of the Indian Council of Agricultural Research (ICAR) Complex, Tripura Centre, Lembucherra, West Tripura, India located at 22°56/N latitude and 90°09/E longitude. During the 60 days experimental period, meteorological data were daily recorded. The climate was cold and humid with environmental minimum temperature ranging between 7.2°C and 15°C and 18.5°C and 25.5°C during the periods of 1^st^-30^th^ and 31^st^-60^th^ day, respectively. The temperature humidity index per day was calculated according to Johnson *et al*. [12] and presented in Figure-1.

### Animals and management

**Figure 2:**
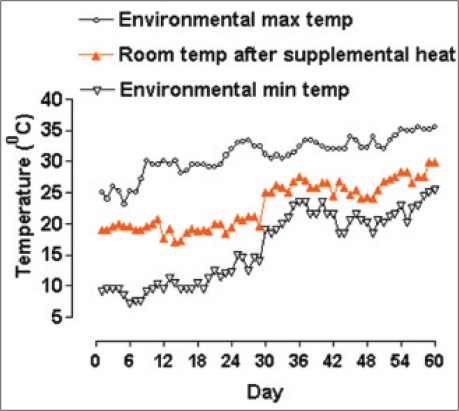
Daily temperature values during the 60 days experimental periods (ambient temperature ranged between 7.2°C and 15.0°C and 18.5°C and 25.5°C for the period of 1^st^-30^th^ and 31^st^-60^th^ day of age, respectively. In case of supplemental heat, values ranged between 17.0°C and 21.1°C and 24.1°C and 29.9°C for the period of 1^st^-30^th^ and 31^st^-60^th^ day of age, respectively).

About 38 indigenous Ghungroo piglets were randomly selected from 4 lactating sows one day after their birth. Each sow with its litter was housed in well-ventilated individual pens with brick flooring and asbestos roofing. The supplemental heat was provided to 19 piglets (10 piglets from sow no. 2549 and 9 piglets from sow no. 2531) by placing three 100 W bulbs 3 ft high from the floor for each pen. Temperature values of the heat supplemented pens ranged from 17.0°C to 21.1°C for the first 30 days period and between 24.1°C and 29.9°C for the next 30 days. These piglets were considered as the treatment group. Another 19 piglets (11 piglets from sow no. 2541 and 8 piglets from sow no. 2546) were housed in separate two pens under natural environmental conditions and considered as the control group. The environmental minimum and maximum temperature along with room temperature after supplemental heat are shown in Figure-2.

Fresh and clean water was offered *ad libitum* by a water trough and piglets had also free access to suckle. They were treated with iron dextran (Imferon^®^, M/s. Shreya, India) intramuscularly at 3, 7, and 14 days of age and vaccinated with swine fever vaccine at 45 days of age. Piglet mortality rates were daily recorded during the experimental period.

### Body weight and rectal temperature recording

Body weight of each piglet was recorded on the day of birth and then on a weekly basis up to 56 days of age. Rectal temperature was also recorded on the day of birth and then at 3 days interval for 60 days.

### Blood sampling

Each piglet was restrained in dorsoventral position and blood sample was collected into heparinized 5 ml polypropylene tubes (20 IU heparin/ml of blood) from anterior vena cava under aseptic condition using 18 gauge needle between 09:30 and 10.30 h on day 7, 15, 30, 45, and 60 of age. A fraction of blood sample was used for hemoglobin estimation using standard Sahli’s acid hematin method [13]. Plasma samples were collected after centrifugation at 2500 × *g* for 10 min at 4°C and stored at −20°C until the implementation of plasma biochemical analyzes.

### Biochemical profiling

Plasma glucose, alkaline phosphatase (AP), glutamate pyruvate transaminase (GPT), and glutamate oxaloacetate transaminase (GOT) activities were estimated colorimetrically using commercially available kits (M/s. Span Diagnostic Ltd., Surat, India). Plasma cortisol, triiodothyronine (T3), thyroxine (T4), follicle-stimulating hormone (FSH), and luteinizing hormone (LH) were quantified by an enzyme-linked immunosorbent assay technique using the commercially available kit for swine (M/s. Endocrine Technologies, Inc., Newark, CA, USA).

### Statistical analysis

Data are presented as the mean±standard error of the mean. The mean±standard error of the mean of different parameters studied were graphically presented using graph pad PRISM 2.01 Software Package (1995). The effect of treatment and period (week/day) on body weight and biochemical parameters was determined by performing an ANOVA analysis appropriate for repeated measures using the SPSS Statistical Software Package (1999), SPSS, Inc., USA.

## Results and Discussion

### Mortality rate

**Figure 3:**
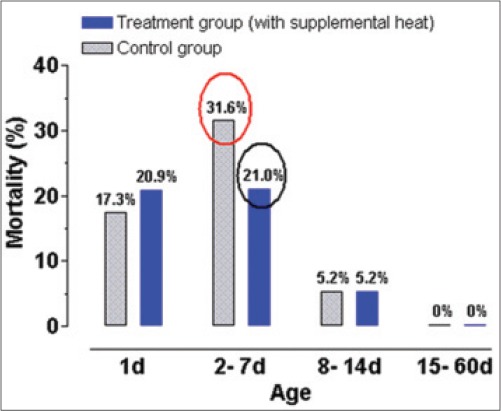
Piglet mortality (%) of control and supplemental heat treated piglets during the experimental period.

The rates of piglet mortality in control and supplementary heat treated piglet groups are presented in Figure-3. In the present experiment, mortality rates of 17.3% and 20.9% were observed at the day of birth in control and treatment groups, respectively, due to stillbirth, asphyxia, low birth weight, crushing, etc. The period between the 2^nd^ and 7^th^ day of age, mortality rates were 31.6% and 21.0% in control and heat supplemented groups, respectively.

This finding may be comparable to earlier observations [14]. Piglet mortality was invariably high in the first few days after birth, reflecting the problems of transition from the totally protected intrauterine life to an unpredictable extrauterine existence.

### Rectal temperature

**Figure 4:**
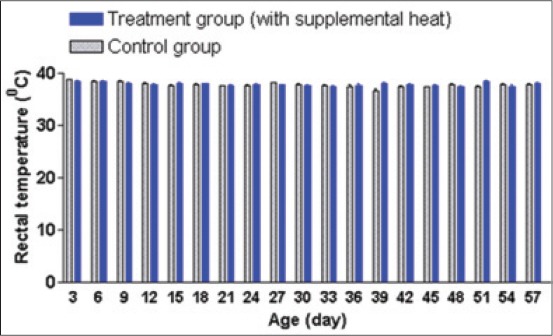
Mean±standard error of mean rectal temperature of control and supplemental heat treated piglets during the experimental period.

Figure-4 shows that the mean rectal temperature in both control and supplemental heat treated piglets remain between 37.31°C and 38.83°C indicating the maintenance of a normothermic temperature of 38-39°C [5].

### Body weight

**Figure 5:**
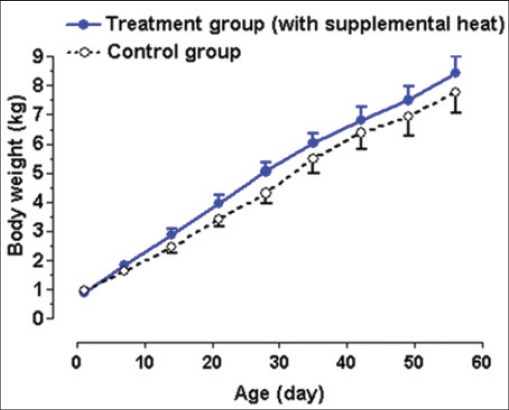
Mean±standard error of mean body weight of control and supplemental heat treated piglets during the experimental period.

The mean±standard error of the mean (SEM) body weight values is presented in Figure-5. No effect (p>0.05) of supplemental heat on body weight was shown, and the daily weight gain for both groups was approximately 130 g/day. This finding is in agreement with the data of exotic piglets, which were reared under an artificial temperature between 18.5°C and 22.5°C [14]. In contrast, Adams *et al*. [15] reported that supplemental heat improved weight gain, while pigs were housed in farrowing crates with 250-watt lamp and the ambient temperature of the farrowing house was approximately 21°C at sow’s level. Weight is considered as the most important factor in successful recovery from postnatal hypothermia [16].

### Plasma biochemical profiles

**Figure 6:**
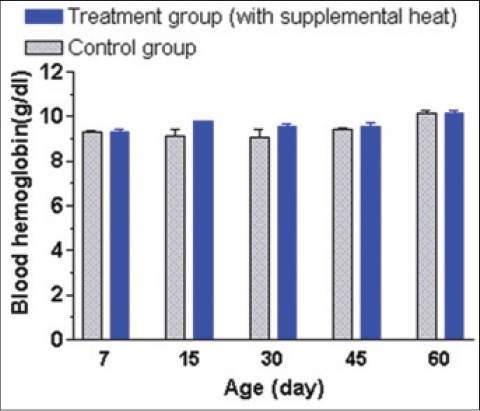
Mean±standard error of mean blood hemoglobin levels (g/dl) of control and supplemental heat treated piglets during the experimental period.

**Figure 7:**
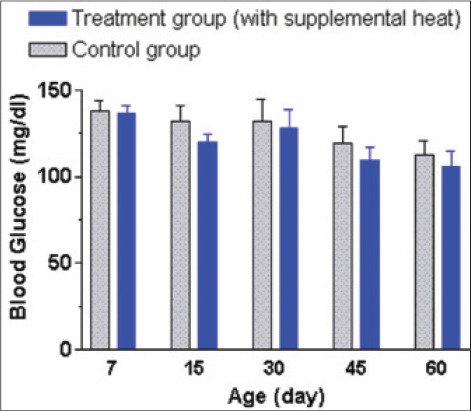
Mean±standard error of mean plasma glucose levels (mg/dl) of control and supplemental heat treated piglets during the experimental period.

The mean±SEM blood hemoglobin and glucose concentrations of the supplemental heat treated and the control piglets recorded at 7, 15, 30, 45, and 60 days of age are presented in Figures-6 and 7, respectively. There was no significant effect (p>0.05) of the supplemental heat on the levels of these blood parameters. The mean blood hemoglobin and glucose concentrations in both groups were comparable with the values reported in 6-8 months old indigenous Assam pigs [17] and weaned Burmese pigs [18]. Recent reports indicated that maternal dietary protein level induced changes in the epigenetic regulation of the glucose metabolism [19] and microRNA involved lipid metabolism [20] in newborn piglet liver. Other reports indicated that plasma glucose level increased linearly when newborn piglets were exposed to the temperature of 14°C for 2-2.5 h [5]. The gradual decrease in plasma glucose concentration with the advancement of age in both experimental groups could be a result of the increase in environmental temperature as the experiment continued.

**Figure 8:**
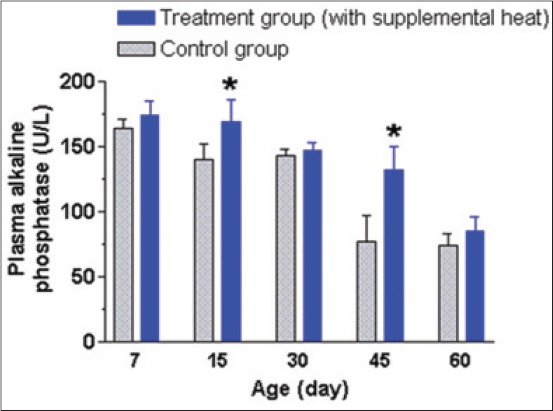
Mean±standard error of mean plasma alkaline phosphatase levels (U/L) of control and supplemental heat treated piglets during the experimental period (*p<0.05).

**Figure 9:**
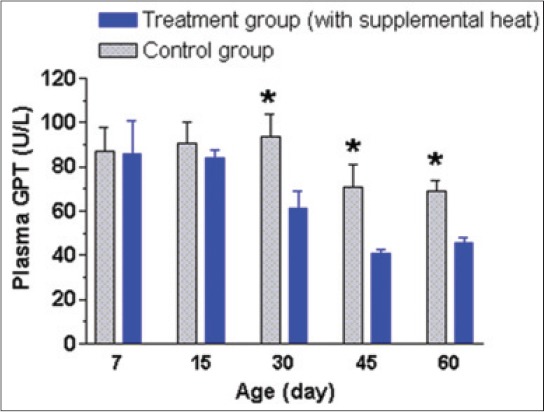
Mean±standard error of mean plasma glutamate pyruvate transaminase levels (U/L) of control and supplemental heat treated piglets during the experimental period (*p<0.05).

**Figure 10:**
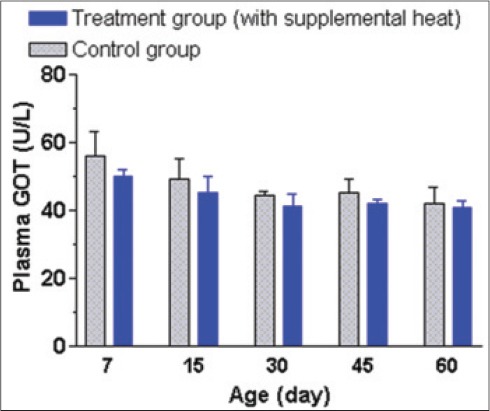
Mean±standard error of mean plasma glutamate oxaloacetate transaminase levels (U/L) of control and supplemental heat treated piglets during the experimental period.

**Figure 11:**
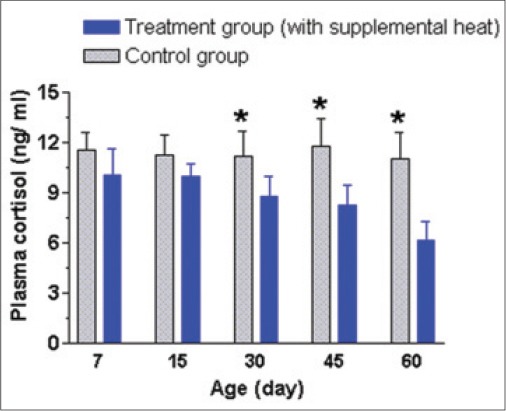
Mean±standard error of mean plasma cortisol concentrations (ng/ml) of control and supplemental heat treated piglets during the experimental period (*p<0.05).

The mean±SEM plasma AP, GPT, GOT, and cortisol levels of the supplemental heat treated and the control piglets at 7, 15, 30, 45, and 60 days of age presented in Figures-8-11, respectively. Plasma AP and GPT concentration gradually decreased (p<0.05) with age in both groups (Figures-8 and 9). This decrease of plasma AP with the advancement of age in both groups indicated that the higher activity of AP possibly had a positive effect on mineralization process, osteoblastic activity, and rapid growth process of bone at the early age. In addition, the supplemental heat possibly improved bone growth process in the heat supplemented group as plasma AP concentration was significantly higher (p<0.05) compared to that of the control group at the age of 15 and 45 days (Figure-8). The supplemental heat might also have some beneficial effects on reducing protein metabolism leading to lower (p<0.05) plasma GPT concentration in the treatment compared to the control group at the age of 30, 45, and 60 days (Figure-9). Plasma GOT levels did not vary (p>0.05) between the control and treatment groups during the experimental period (Figure-10). The mean plasma GPT and GOT levels in control and treatment groups are within the range of previously reported values [21]. The increased levels of plasma GPT during the first 2 weeks of life might be an effect of the interaction of early age with the environmental low temperature [22]. On the other hand, the gradual decrease (p<0.05) in plasma GPT at the age of 45 and 60 days in both groups possibly indicated a gradual physiological adjustment to the environmental conditions. As it is presented in Figure-11, there was a gradual decrease of plasma cortisol concentration only in supplemental heat treated piglets. The supplemental heat resulted in lower (p<0.05) plasma cortisol levels on day 30, 45, and 60, as previously observed in newborn pigs on day 2 of age [14]. Possible explanation is that the supplemental heat reduced the stress of young piglets that already possess a functional hypothalamic-pituitary-adrenocortical axis.

**Figure 12:**
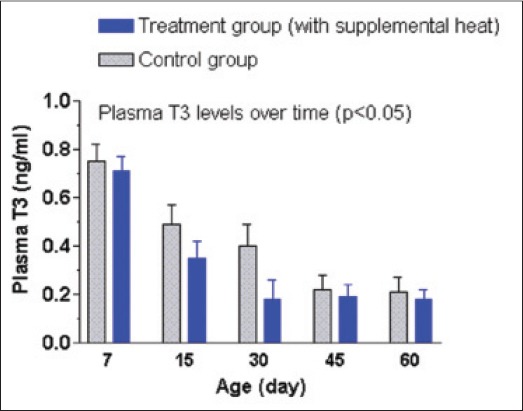
Mean±standard error of mean plasma triiodothyronine concentrations (ng/ml) of control and supplemental heat treated piglets during the experimental period.

**Figure 13:**
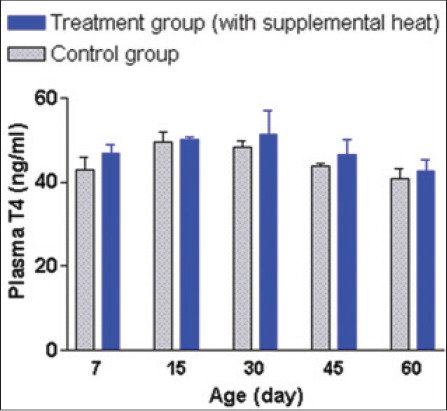
Mean±standard error of mean plasma thyroxine concentrations (ng/ml) of control and supplemental heat treated piglets during the experimental period.

**Figure 14:**
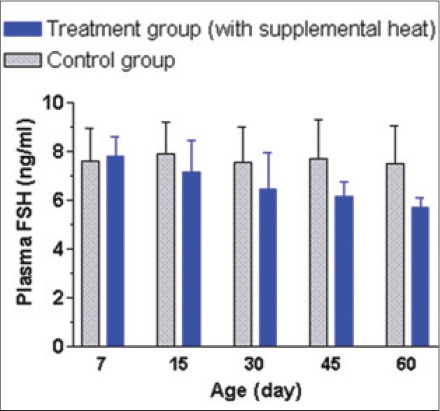
Mean±standard error of mean plasma follicle-stimulating hormone concentrations (ng/ml) of control and supplemental heat treated piglets during the experimental period.

**Figure 15:**
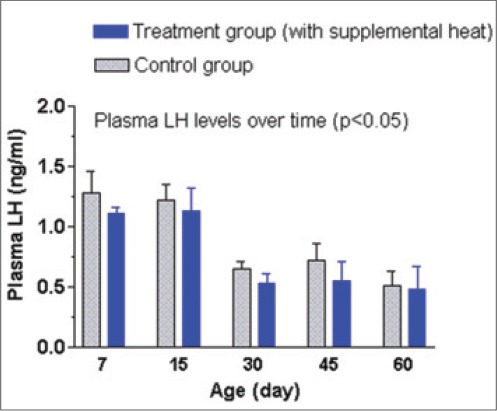
Mean±standard error of mean plasma luteinizing hormone concentrations (ng/ml) of control and supplemental heat treated piglets during the experimental period.

The mean±SEM plasma T3, T4, FSH, and LH concentrations of the supplemental heat treated and the control piglets recorded at 7, 15, 30, 45, and 60 days of age are shown in Figures-12-15, respectively. No significant effect (p>0.05) of the supplemental heat on plasma T3, T4, FSH, and LH concentrations was demonstrated in the present study. However, plasma T3 and LH concentrations decreased (p<0.05) gradually with the advancement of age in both groups.

The present findings on plasma T3 and T4 concentrations in experimental piglets support the observations recorded earlier in neonatal pigs exposed to cold temperatures [23]. Evidence of an increase in the release of thyroid-stimulating hormone (TSH) from the pituitary gland and thereby increase in secretion of the thyroid hormones from the thyroid gland in animals exposed to cold have well been documented [24]. No significant difference (p>0.05) of plasma T4 concentrations between the experimental groups or the gradual decrease (p<0.05) in plasma T3 concentrations with the advancement of age could be explained in the light of adjustments in metabolism and energy expenditure [25]. The mean plasma FSH and LH concentration in control and treatment groups were quite similar with the concentrations recorded in Landrace x Yorkshire crossbred neonatal pigs [26]. The gradual decrease (p<0.05) in plasma LH concentration with the advancement of age in both groups might be due to the ovarian steroid negative feedback mechanism on gonadotropin secretion in neonatal piglets [27].

## Conclusions

To the best of the authors’ knowledge, the present study is the first that describes the effect of supplemental heat on mortality rate, growth performance, and blood biochemical profiles of indigenous Ghungroo piglets during cold and humid weather in north eastern hill of India. The supplemental heat resulted in approximately 10% reduction of piglet mortality during the first week of life under farm management system in the sub-tropical cold and humid climatic conditions of north eastern region of India. However, the observations on the effect of supplemental heat on piglet performance warrant validation on different breeds of piglets. The effect of supplemental heat at higher temperatures on piglet performances also needs to be examined.

## Authors’ Contributions

All authors contributed to conception and design of the study. HN and MH worked together at the farm as well as research laboratory to collect data. MD analyzed data. DR monitored the whole research program. AH interpreted the results and drafted the article critically for important intellectual content. All authors read and approved the final manuscript.

## References

[1] Kirkden R.D, Broom D.M, Andersen I.L (2013). Invited review: Piglet mortality: Management solutions. J. Anim. Sci.

[2] Strange T, Ask B, Nielsen B (2013). Genetic parameters of the piglet mortality traits stillbirth, weak at birth, starvation, crushing and miscellaneous in crossbred pigs. J. Anim. Sci.

[3] Herpin P, Damon M, Le Dividich J (2002). Development of thermoregulation and neonatal survival in pigs. Livest. Prod. Sci.

[4] Berthon D, Herpin P, Bertin R, De Marco F, le Dividich J (1996). Metabolic changes associated with sustained 48-hr shivering thermogenesis in the newborn pig. Comp. Biochem. Physiol. B Biochem. Mol. Biol.

[5] Lossec G, Herpin P, Le Dividich J (1998). Thermoregulatory responses of the newborn pig during experimentally induced hypothermia and rewarming. J. Exp. Physiol.

[6] Alonso-Spilsbury M, Mota-Rojas D, Villanueva-Garcia D, Martines-Burnes J, Orozco H, Ramirez-Necoechea R, Lopez M.A, Truijillo M.E (2005). Perinatal asphyxia pathophysiology and human: A review. Anim. Reprod. Sci.

[7] Zhong X, Li W, Huang X, Zhang L, Yimamu M, Raiput N, Zhou Y, Wang T (2012). Impairment of cellular immunity is associated with overexpression of heat shock protein 70 in neonatal pigs with intrauterine growth retardation. Cell Stress Chaperones.

[8] Pedersen L.J, Malmkvist J, Kammersgaard T, Jørgensen E (2013). Avoiding hypothermia in neonatal pigs: Effect of duration of floor heating at different room temperatures. J. Anim. Sci.

[9] Westin R, Holmgren N, Hultgren J, Ortman K, Linder A, Algers B (2015). Post-mortem findings and piglet mortality in relation to strategic use of straw at farrowing. Prev. Vet. Med.

[10] Cai D, Jia Y, Song H, Sui S, Lu J, Jiang Z, Zhao R (2014). Betaine supplementation in maternal diet modulates the epigenetic regulation of hepatic gluconeogenic genes in neonatal piglets. PLoS One.

[11] Kenneth B (1986). Bioenergetics and growth: The whole and the parts. J. Anim. Sci.

[12] Johnson H.D, Ragsdale A.C, Berry I.L, Shanklin M.D (1963). Temperature humidity effects including influence of acclimation feed and water consumption of Holstein cattle.

[13] Sahli H (1909). Untersuchungen Methode.

[14] McGinnis R.M, Marple D.N, Ganjam V.K, Prince T.J, Pritchett J.F (1981). The effect of floor temperature, supplemental heat and drying at birth on neonatal swine. J. Anim. Sci.

[15] Adams K.L, Baker T.H, Jensen A.H (1980). Effect of supplemental heat for nursing piglets. J. Anim. Sci.

[16] Kammersgaard T.S, Pedersen L.J, Jørgensen E (2011). Hypothermia in neonatal piglets: Interactions and causes of individual differences. J. Anim. Sci.

[17] Phookan A, Laskar S, Goswami R.N, Deori S (2011). Hemoglobin type, hemoglobin concentration and serum alkaline phosphatase level in indigenous pigs of Assam. Tamilnadu J. Vet. Anim. Sci.

[18] Sarma K, Konwar B, Ali A (2011). Hemato-biochemical parameters of Burmese pig of subtropical hill agro ecosystem. Indian J. Anim. Sci.

[19] Jia Y, Cong R, Li R, Yang X, Sun Q, Parvizi N, Zhao R (2012). Maternal low-protein diet induces gender-dependent changes in epigenetic regulation of the glucose-6-phosphatase gene in newborn piglet liver. J. Nutr.

[20] Pan S, Zheng Y, Zhao R, Yang X (2013). MicroRNA-130b and microRNA-374b mediate the effect of maternal dietary protein on offspring lipid metabolism in Meishan pigs. Br. J. Nutr.

[21] Dhanotiya R.S (2006). Textbook of Veterinary Biochemistry.

[22] Nirupama R, Devaki M, Yajurvedi H.N (2010). Repeated acute stress induced alternations in carbohydrate metabolism in rats. J. Stress Physiol. Biochem.

[23] Macari M, Dauncey M.J, Ramsden D.B, Ingram D.Z (1983). Thyroid hormone metabolism after acclimatization to a warm or cold temperature under the condition of high or low energy intake. J. Exp. Physiol.

[24] Macari M, Zuim S.M, Secato E.R, Guerreiro J.R (1986). Effect of ambient temperature and thyroid hormone on food intake by pigs. J. Physiol. Behav.

[25] Laurberg P, Anderson S, Kermisolt J (2005). Cold adaptation and thyroid hormone metabolism: Review. Horm. Metab. Res.

[26] Colenbrander B, Meijer J.C, Macdonald A.A, Van De Wiel D.F.M, Engel B, De Jong F.H (1987). Feedback regulation of gonadotropic hormone secretion in neonatal pigs. Biol. Reprod.

[27] Campbell C.S, Schwartz M.B (1977). Steroid feedback regulation of luteinizing hormone and follicle - Stimulating hormone secretion rates in male and females rats. J. Toxicol. Environ. Health.

